# Neutrophil-to-lymphocyte ratio as a harbinger of peritonitis in peritoneal dialysis: a case–control study

**DOI:** 10.3389/fmed.2026.1787005

**Published:** 2026-04-16

**Authors:** Wenhui Lei, Hai-ping Lai, Xiaoli Sun, Jun Xin

**Affiliations:** 1 Nephrology and Urology Center, Fifth Affiliated Hospital of Wenzhou Medical University, Lishui, Zhejiang, China; 2 Department of Rheumatology and Immunology, The Fifth Affiliated Hospital of Wenzhou Medical University, Lishui, Zhejiang, China; 3 Department of Medicine, Ganzhou Cancer Hospital, Ganzhou, Jiangxi, China; 4 Department of Medicine, The First Hospital of Quanzhou Affiliated to Fujian Medical University, Quanzhou, Fujian, China

**Keywords:** biomarker, inflammation, neutrophil-to-lymphocyte ratio, peritoneal dialysis, peritonitis, risk factor

## Abstract

**Background:**

The neutrophil-to-lymphocyte ratio (NLR) is a biomarker of systemic inflammation and has been associated with adverse outcomes in dialysis patients. Its role in predicting the future risk of peritoneal dialysis-associated peritonitis (PDAP) before clinical onset remains unclear.

**Objective:**

To evaluate the association between pre-peritonitis NLR levels and the risk of developing PDAP.

**Methods:**

We conducted a retrospective, matched (1:1) case–control study involving patients on peritoneal dialysis at a single center between January 2010 and October 2024. Cases were patients who developed a first episode of PDAP (diagnosed per ISPD guidelines). Controls were matched to cases on sex and age (±3 years). The exposure was NLR measured from routine blood tests during a 3-month period preceding the peritonitis event for cases, or a corresponding pre-index period for controls. Logistic regression analysis was used to assess the association.

**Results:**

A total of 178 patients (89 cases and 89 matched controls) were included in the analysis. In conditional logistic regression models accounting for the matched design, a higher NLR was independently associated with an increased risk of PDAP. When analyzed as a continuous variable (per 1-unit increase in ln(NLR)), the fully adjusted odds ratio (OR) was 2.25 (95% confidence interval [CI]: 1.26–4.02, *p* = 0.006). When NLR was categorized into tertiles, patients in the highest tertile (NLR ≥ 1.24) demonstrated a consistent positive association with peritonitis risk compared to those in the lowest tertile (NLR < 0.52), with a fully adjusted OR of 11.00 (95% CI: 3.06–39.52, *p* < 0.001; P for trend < 0.001). Restricted cubic spline analysis revealed a significant linear dose–response relationship (P for non-linearity = 0.511). An elevated NLR (≥ median) also showed moderate discriminative ability for predicting PDAP, with an area under the curve (AUC) of 0.78 (95% CI: 0.71–0.85).

**Conclusion:**

A higher NLR measured during a clinically stable period prior to infection is associated with an increased risk of subsequent PDAP. If validated in prospective studies, NLR may serve as a simple and readily available biomarker for risk stratification in this population.

## 1 Introduction

Peritoneal dialysis (PD) is a widely utilized renal replacement therapy for end-stage renal disease. However, its clinical course is frequently complicated by peritoneal dialysis-associated peritonitis (PDAP), a major cause of morbidity, technique failure, and mortality in this population, with mortality rates ranging from 2 to 6% (1). Beyond acute morbidity, recurrent peritonitis episodes and the chronic inflammatory state inherent to PD patients contribute to peritoneal membrane damage, fibrosis, and ultimately, PD discontinuation. Therefore, identifying modifiable risk factors for PDAP is crucial for improving patient outcomes and preserving peritoneal membrane function.

The neutrophil-to-lymphocyte ratio (NLR), a readily calculated hematologic index derived from routine complete blood counts, has emerged as a robust and accessible biomarker of systemic inflammation and immune dysregulation (2). In the context of end-stage renal disease, elevated NLR has been consistently associated with adverse outcomes. Cross-sectional and cohort studies in hemodialysis (HD) populations have linked higher NLR to increased systemic inflammation and poor survival (3). Furthermore, recent evidence suggests that NLR measured at the time of diagnosis is predictive of treatment failure in established PDAP (4). However, no studies have examined whether NLR levels prior to the onset of peritonitis are associated with the development of PDAP in patients undergoing PD.

Despite these associations, a critical gap remains in understanding whether a pre-existing, subclinical inflammatory state-as reflected by NLR-predisposes patients to the initial development of peritonitis. Specifically, no studies have investigated the association between NLR levels measured during a stable, infection-free period and the subsequent risk of incident PDAP.

To address this knowledge gap, we conducted a retrospective case–control study. Our primary objective was to evaluate whether an elevated NLR, measured up to 3 months prior to clinical presentation, is associated with an increased risk of developing PDAP in patients undergoing peritoneal dialysis.

## 2 Methods

### 2.1 Study design and setting

We conducted a retrospective, matched case–control study at the Department of Nephrology, The Fifth Affiliated Hospital of Wenzhou Medical University. The study period spanned from January 2010 to October 2024. Data were extracted from electronic medical records between November 2024 and January 2025. The study protocol was approved by the Medical Ethics Committee of the Fifth Affiliated Hospital of Wenzhou Medical University (Approval No: 2025(I)-081–01), which waived the requirement for informed consent due to the retrospective nature of the study.

### 2.2 Participant selection

Case Definition and Identification: We used retrospective consecutive enrollment to identify all first-episode PD-associated peritonitis cases that met the ISPD diagnostic criteria. Cases were adult patients (age ≥18 years) undergoing peritoneal dialysis (PD) who developed their first episode of PD-associated peritonitis (PDAP) during the study period. Peritonitis was diagnosed according to the International Society for Peritoneal Dialysis (ISPD) guidelines, requiring at least two of the following criteria: (1) abdominal pain with cloudy dialysate, with or without fever; (2) dialysate white blood cell count >100 cells/μL with ≥50% polymorphonuclear neutrophils; or (3) positive dialysate culture.

Control Selection: Controls were selected using 1:1 nearest-neighbor greedy matching without replacement, based on sex and age (±3 years) to minimize confounding. For each case, one control was drawn from the pool of PD patients who had not experienced peritonitis by the time of the case’s peritonitis event (the index date). If a suitable match could not be found, the case was excluded from the analysis (5).

**Figure 1:**
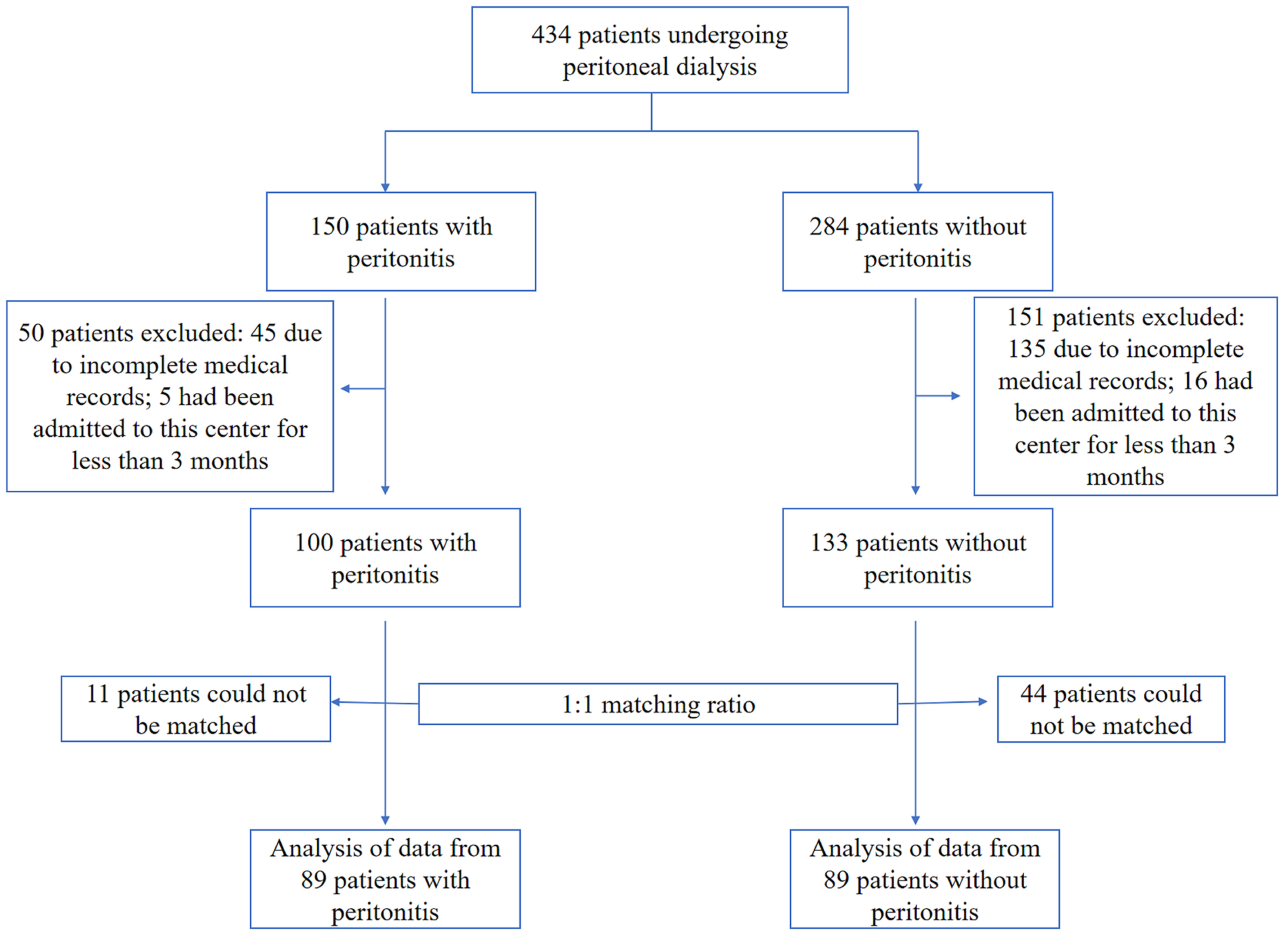
Patient enrollment flowchart.

Inclusion and Exclusion Criteria: For both cases and controls, inclusion required continuous follow-up at our center for at least 3 months prior to the index date and availability of complete baseline clinical and laboratory data. We excluded patients who met any of the following criteria: (1) peritonitis secondary to traumatic injury, intestinal perforation, tunnel/exit-site infection, or biliary tract infection; (2) current use of immunosuppressive therapy or presence of a primary immunodeficiency; (3) diagnosis of any active infectious disease (e.g., pulmonary, urinary tract, gastrointestinal) or non-infectious inflammatory condition (e.g., active autoimmune disease, inflammatory bowel disease flare) within 3 months before the index date. Data extraction was performed independently by two researchers who were blinded to case/control status. The detailed patient selection process is summarized in Figure 1.

### 2.3 Definitions of exposure and covariates

The primary exposure variable was the neutrophil-to-lymphocyte ratio (NLR), calculated as the absolute neutrophil count divided by the absolute lymphocyte count from a single routine peripheral blood test obtained during the predefined 3-month window.

For cases, the exposure NLR was defined as the value measured during a 3-month period immediately preceding the diagnosis of peritonitis (the index date), but excluding any laboratory tests performed after the onset of symptoms (2).

For controls, the exposure NLR was obtained from a blood test performed during a corresponding 3-month window prior to their individually assigned index date. This index date for each control was defined as the date on which they had accumulated the same duration of PD follow-up as their matched case had at the time of peritonitis onset. This approach ensured temporal comparability of exposure assessment between cases and controls.

Baseline covariates were collected from the same pre-index date 3-month window and included: demographic factors (age, sex, education level), clinical characteristics (dialysis vintage, smoking status, history of hypertension, history of diabetes mellitus, primary renal disease, body mass index), and laboratory parameters (hemoglobin, albumin, globulin, serum calcium, blood urea nitrogen, serum creatinine, parathyroid hormone, C-reactive protein, total white blood cell count, and urea clearance index [Kt/V]).

### 2.4 Statistical analysis

Continuous variables are presented as mean ± standard deviation if normally distributed, or as median (interquartile range) if non-normally distributed. Normality was assessed using the Shapiro–Wilk test. Categorical variables are presented as frequencies and percentages. Group comparisons between cases and controls were performed using the independent samples *t*-test or Mann–Whitney *U* test for continuous variables, and the chi-square test or Fisher’s exact test for categorical variables, as appropriate.

The association between NLR and PDAP risk was evaluated using conditional logistic regression to account for the matched design. NLR was analyzed in two ways: (1) as a continuous variable using ln(NLR), and (2) as a categorical variable based on tertiles of the NLR distribution among all participants (Q1: <0.52, Q2: 0.52–1.24, Q3: ≥1.24). The trend across tertiles was tested by modeling the tertile median as a continuous variable (6).

We constructed a series of hierarchical models to assess the robustness of the association with progressive adjustment for potential confounders: Model 1 (Crude): Unadjusted. Model 2: Adjusted for the matching variables (age, sex). Model 3: Model 2 + education level, smoking status, diabetes, hypertension, and dialysis vintage. Model 4 (Fully Adjusted): Model 3 + laboratory covariates: parathyroid hormone, hemoglobin, albumin, calcium, globulin, C-reactive protein, and white blood cell count. Results are reported as odds ratios (ORs) with 95% confidence intervals (CIs). To explore the dose–response relationship, a restricted cubic spline model with three knots was fitted for ln(NLR) in the fully adjusted model, testing for non-linearity.

Sensitivity analyses were conducted in two steps. First, a multivariable, unconditional logistic regression was performed including all variables with *p* < 0.20 in the bivariate analyses. Second, the discriminative performance of the neutrophil-lymphocyte ratio (NLR) was evaluated using receiver operating characteristic (ROC) curve analysis. All statistical analyses were conducted in R (version 4.4.0) and SPSS (version 26.0). A two-tailed *p* value < 0.05 was considered statistically significant. To avoid overfitting, we first applied least absolute shrinkage and selection operator (LASSO) regression for variable selection. Concurrently, a simple model was constructed using core variables identified: ln(NLR), dialysis vintage, albumin, and C-reactive protein (CRP). These core variables were used to develop a simplified model.

## 3 Results

### 3.1 Baseline clinical data of participants

A total of 178 peritoneal dialysis patients (89 cases with peritonitis, 89 controls) were analyzed (Table 1). Compared to controls, cases were younger (median 51.0 vs. 56.0 years; *p* = 0.017) and had shorter dialysis duration (42.0 vs. 59.0 months; *p* < 0.001). They also exhibited higher CRP (median 2.10 vs. 2.00 mg/L; *p* = 0.034), higher BUN (19.80 ± 7.33 vs. 17.56 ± 4.31 mmol/L; *p* = 0.015), lower globulin (29.44 ± 5.24 vs. 31.39 ± 4.43 g/L; *p* = 0.008), higher ln(NLR) (median −0.01 vs. -0.54; *p* < 0.001), and a higher proportion with Ca < 2.0 mmol/L (16.85% vs. 4.49%; *p* = 0.008). No significant differences were found in other baseline characteristics (all *p* > 0.05).

**Table 1:** Baseline characteristics of peritoneal dialysis patients with and without subsequent peritonitis.

Variables	Total (*n* = 178)	Control (*n* = 89)	Case (*n* = 89)	Statistic	*P*
Age, M (Q_1_, Q_3_)	54.00 (47.25, 63.00)	56.00 (49.00, 64.00)	51.00 (46.00, 61.00)	*Z* = −2.38	0.017
Gender, *n* (%)				*χ*^2^ = 0.58	0.448
Female	75 (42.13)	35 (39.33)	40 (44.94)		
Male	103 (57.87)	54 (60.67)	49 (55.06)		
WBC(10^9^/L), Mean ± SD	7.27 ± 2.91	6.98 ± 2.66	7.56 ± 3.13	*t* = −1.33	0.186
CRP, M (Q_1_, Q_3_)	2.00 (1.00, 5.00)	2.00 (1.00, 3.00)	2.10 (1.00, 7.00)	*Z* = −2.11	0.034
Hemoglobin(g/L), Mean ± SD	105.89 ± 17.65	107.61 ± 14.20	104.18 ± 20.48	*t* = 1.30	0.196
Albumin(g/L), Mean ± SD	36.49 ± 4.67	36.95 ± 3.59	36.03 ± 5.52	*t* = 1.30	0.194
Globulin(g/L), Mean ± SD	30.42 ± 4.93	31.39 ± 4.43	29.44 ± 5.24	*t* = 2.68	0.008
Phosphorus(mmol/L), Mean ± SD	1.58 ± 0.50	1.55 ± 0.49	1.61 ± 0.52	*t* = −0.84	0.405
BUN(mmol/L), Mean ± SD	18.68 ± 6.10	17.56 ± 4.31	19.80 ± 7.33	*t* = −2.47	0.015
CR(umol/L), Mean ± SD	982.72 ± 301.51	1003.36 ± 283.11	962.09 ± 319.13	*t* = 0.91	0.363
ln(NLR), M (Q_1_, Q_3_)	−0.44 (−0.61, 0.16)	−0.54 (−0.66, −0.42)	−0.01 (−0.48, 1.42)	*Z* = −6.50	<0.001
Dialysis duration (Month), M (Q_1_, Q_3_)	49.50 (27.00, 71.75)	59.00 (40.00, 79.00)	42.00 (18.00, 61.00)	*Z* = −3.78	<0.001
Primary disease, *n* (%)				–	0.213
Chronic glomerulonephritis	108 (60.67)	48 (53.93)	60 (67.42)		
Hypertension	63 (35.39)	37 (41.57)	26 (29.21)		
Diabetes or other	7 (3.93)	4 (4.49)	3 (3.37)		
Smoking, *n* (%)				*χ*^2^ = 0.03	0.865
No	131 (73.60)	66 (74.16)	65 (73.03)		
Yes	47 (26.40)	23 (25.84)	24 (26.97)		
Education level, *n* (%)				*χ*^2^ = 3.25	0.197
Illiterate	32 (17.98)	14 (15.73)	18 (20.22)		
Below junior high school	62 (34.83)	27 (30.34)	35 (39.33)		
At or above junior high school	84 (47.19)	48 (53.93)	36 (40.45)		
Diabetes, *n* (%)				*χ*^2^ = 2.07	0.150
No	165 (92.70)	85 (95.51)	80 (89.89)		
Yes	13 (7.30)	4 (4.49)	9 (10.11)		
Hypertension, *n* (%)				*χ*^2^ = 1.50	0.220
No	43 (24.16)	18 (20.22)	25 (28.09)		
Yes	135 (75.84)	71 (79.78)	64 (71.91)		
PTH (pg/ml), *n* (%)				*χ*^2^ = 0.80	0.370
PTH < 600	130 (73.45)	68 (76.40)	62 (70.45)		
PTH ≥ 600	47 (26.55)	21 (23.60)	26 (29.55)		
Ca (mmol/L), *n* (%)				*χ*^2^ = 7.13	0.008
Ca < 2.0	19 (10.67)	4 (4.49)	15 (16.85)		
Ca ≥ 2.0	159 (89.33)	85 (95.51)	74 (83.15)		

BUN, blood urea nitrogen; CRP, C-reactive protein; M, Median; NLR, neutrophil-to-lymphocyte ratio; PTH, parathyroid hormone; Q_1_, 1st quartile; Q₃, 3rd quartile; SD, standard deviation; WBC, white blood cell count; *t*, *t*-test, Z, Mann–Whitney test, *χ*^2^, Chi-square test, −, Fisher exact; SD, standard deviation, M, Median, Q_1_, 1st Quartile, Q_3_, 3st Quartile.

### 3.2 Association between neutrophil-to-lymphocyte ratio and peritoneal Dialysis-associated peritonitis

The association between NLR and PDAP risk was first evaluated using conditional logistic regression accounting for the matched pairs. As shown in Table 2, when analyzed as a continuous variable (per 1-unit increase in ln(NLR)), NLR showed a trend toward increased peritonitis risk in the unadjusted model (OR 1.83, 95% CI 0.97–3.46, *p* = 0.062). After adjustment for the matching variables (age and sex) in Model I, the association became statistically significant (OR 2.13, 95% CI 1.17–3.89, *p* = 0.014). This positive association remained robust with further adjustment for additional demographic and clinical covariates (Model II: OR 2.32, 95% CI 1.29–4.15, *p* = 0.005) and in the fully adjusted model incorporating key laboratory parameters (Model III: OR 2.25, 95% CI 1.26–4.02, *p* = 0.006).

**Table 2:** Multivariable logistic regression analysis of NLR and peritoneal dialysis-related peritonitis.

Variable	n.total	n.event_%	Crude model		Model I		Model II		Model III	
			OR (95%CI)	*p* value	OR (95%CI)	*p* value	OR (95%CI)	*p* value	OR (95%CI)	*p* value
NLR (continuous)	178	89 (50)	1.83 (0.97–3.46)	0.062	2.13 (1.17–3.89)	0.014	2.32 (1.29–4.15)	0.005	2.25 (1.26–4.02)	0.006
NLR (categorical)										
Q1 (<0.52)	59	22 (37.3)	1(Ref)		1(Ref)		1(Ref)		1(Ref)	
Q2 (0.52–1.24)	59	21 (35.6)	0.93 (0.44–1.97)	0.848	0.85 (0.39–1.83)	0.672	0.79 (0.34–1.82)	0.577	0.99 (0.38–2.54)	0.981
Q3 (≧1.24)	60	46 (76.7)	5.53 (2.49–12.27)	<0.001	7.41 (2.93–18.76)	<0.001	9.13 (3.37–24.69)	<0.001	11 (3.06–39.52)	<0.001
P for trend				<0.001		<0.001		<0.001		<0.001

OR, odds ratio; CI, confidence interval; NLR, neutrophil-to-lymphocyte ratio.

Crude model: unadjusted.

Model I: Adjusted for sex and age.

Model II: Adjusted for sex, age, education level, smoking, diabetes, hypertension, and dialysis duration.

Model III: Fully adjusted for sex, age, education level, smoking, diabetes, hypertension, dialysis duration, PTH, hemoglobin, albumin, calcium, globulin, CRP, and WBC.

When NLR was categorized into tertiles, patients in the highest tertile (NLR ≥ 1.24) had markedly increased odds of PDAP compared to those in the lowest tertile (NLR < 0.52). In the fully adjusted model (Model III), the odds ratio for the highest tertile was 11.00 (95% CI 3.06–39.52, *p* < 0.001). A significant dose–response relationship was evident across increasing tertiles (P for trend < 0.001).

**Figure 2:**
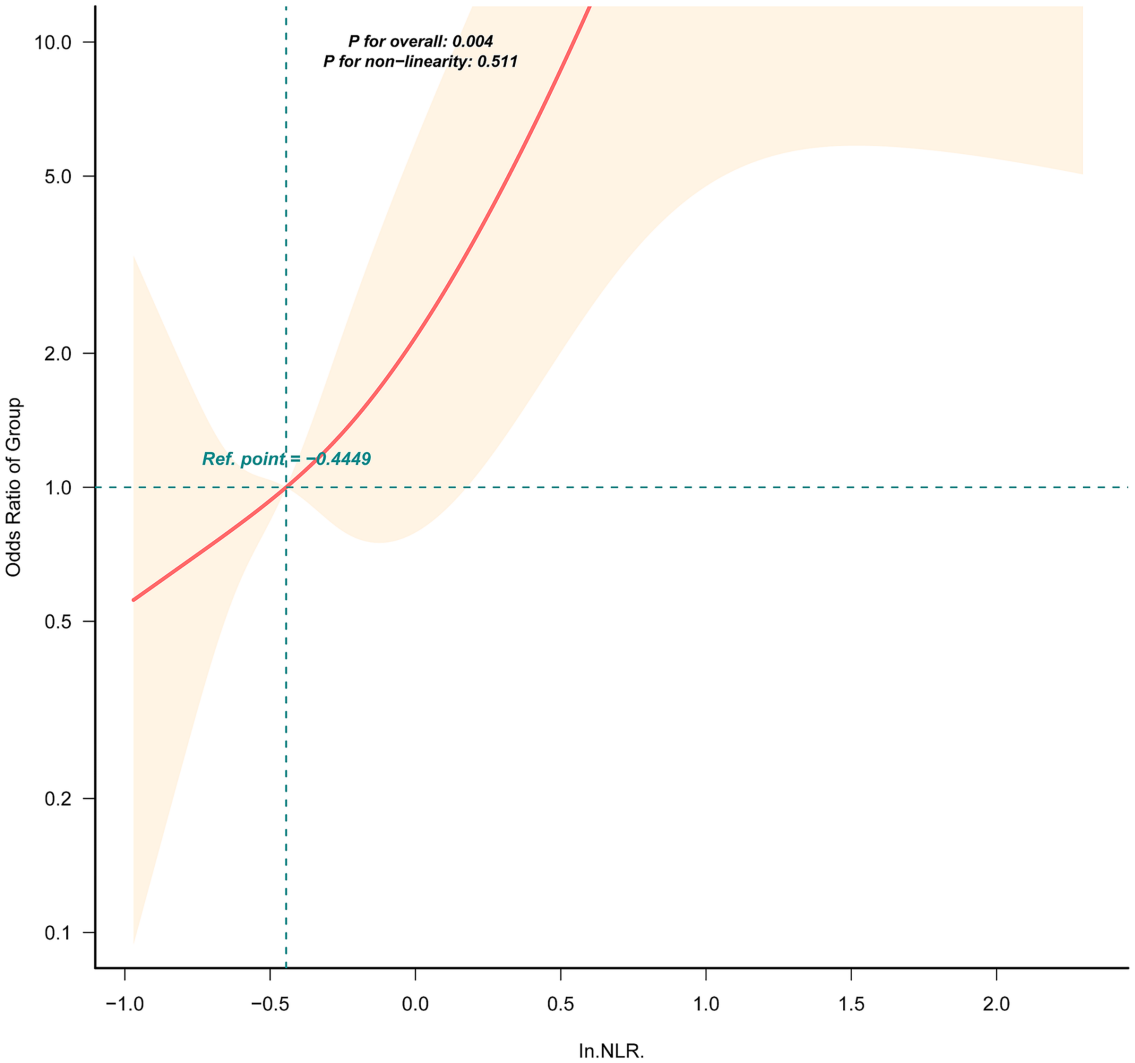
Restricted cubic spline analysis showing the relationship between neutrophil-to-lymphocyte ratio (NLR) and the risk of peritoneal dialysis-associated peritonitis (PDAP). Models were adjusted for sex, age, education level, smoking, diabetes, hypertension, dialysis duration, parathyroid hormone (PTH), hemoglobin, albumin, calcium, globulin, C-reactive protein (CRP), and white blood cell count (WBC).

To visualize the dose–response relationship, a restricted cubic spline analysis was performed. As illustrated in Figure 2, a significant linear association was observed between ln(NLR) and the log odds of peritonitis (P for overall association = 0.004; P for non-linearity = 0.511), confirming a monotonically increasing risk with higher NLR levels.

### 3.3 Subgroup analysis

**Figure 3:**
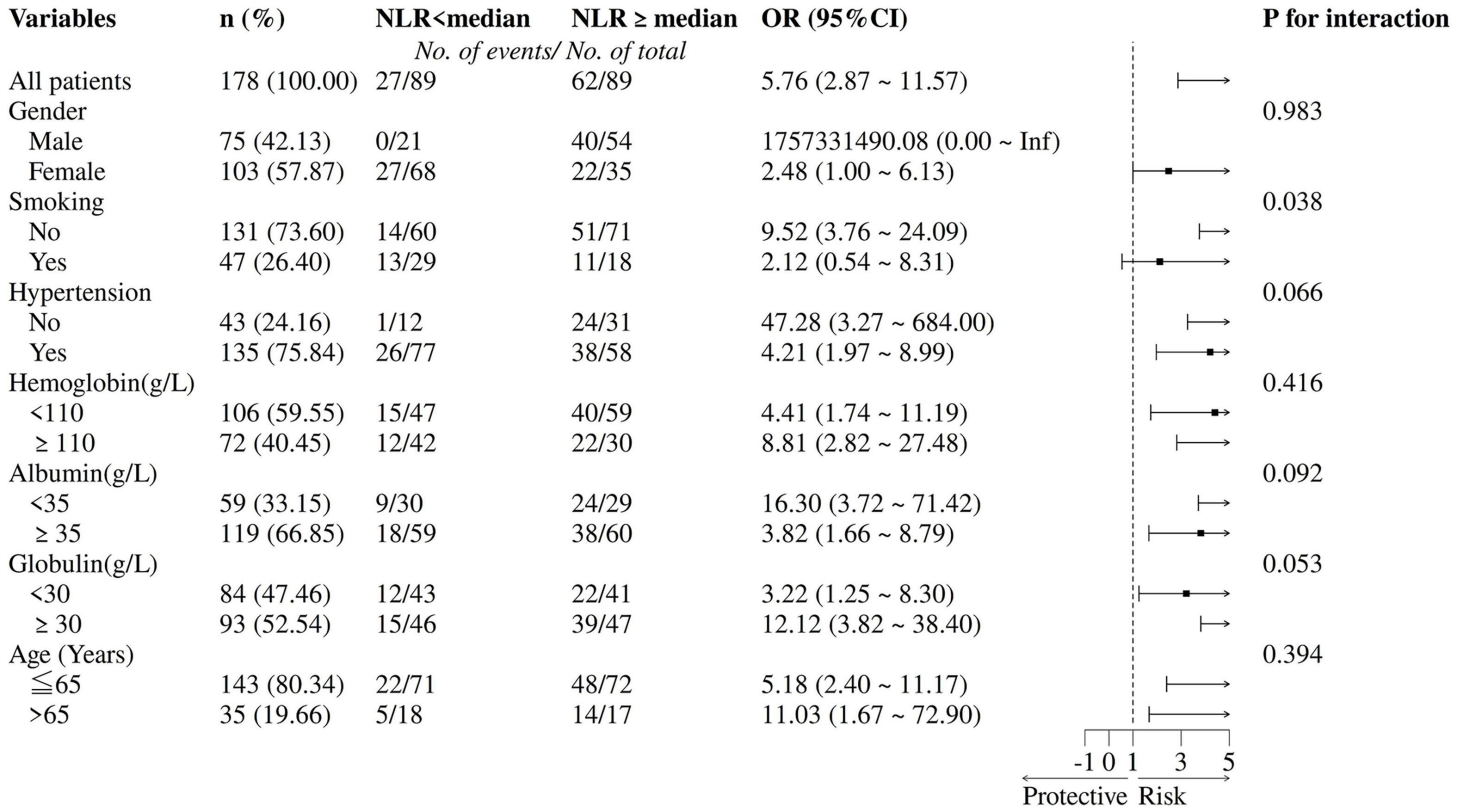
Subgroup analysis of the association between NLR and the risk of peritonitis. NLR was dichotomized at the median value. Multivariable logistic regression was performed to assess the relationship between NLR categories and peritonitis incidence.

Stratified analyses demonstrated a consistent positive association between elevated NLR (≥ median) and PDAP risk across most clinical subgroups. In the overall cohort, an elevated NLR was associated with significantly increased odds of peritonitis (OR: 5.76; 95% CI: 2.87–11.57) (Figure 3). A statistically significant interaction was observed for gender (P for interaction = 0.038). Consistently positive associations were also observed in several high-risk subgroups, including patients without hypertension (OR: 47.28; 95% CI: 3.27–684.00), those with albumin <35 g/L (OR: 16.30; 95% CI: 3.72–71.42), and those with globulin ≥30 g/L (OR: 12.12; 95% CI: 3.82–38.40). However, the wide confidence intervals in these subgroups reflect limited sample sizes and should be interpreted with caution. No significant interactions were detected for hypertension, hemoglobin, albumin, globulin, or age (all P for interaction >0.05), suggesting that the association between NLR and PDAP risk remains broadly consistent across patient profiles. Given the exploratory nature of these analyses and the modest sample size in certain strata, these findings warrant confirmation in larger, adequately powered studies.

### 3.4 Sensitivity analysis

To assess the robustness of our primary finding, we performed complementary sensitivity analyses. First, we employed an alternative modeling strategy using unconditional multivariable logistic regression and entering all variables with *p* < 0.20 from bivariate analysis into a single model. In this analysis, NLR remained an independent predictor of PDAP (adjusted OR 3.01, 95% CI 1.23–7.33, *p* = 0.015) (Supplementary Table S1).

Second, we evaluated the discriminative ability of NLR alone for predicting peritonitis using receiver operating characteristic (ROC) curve analysis. The area under the ROC curve (AUC) was 0.78 (95% CI 0.71–0.85) (Supplementary Figure S1), indicating moderate discriminatory power. At the optimal cutoff value of 0.459, sensitivity was 0.85 (95% CI 0.78–0.93) and specificity was 0.65 (95% CI 0.55–0.75) (Supplementary Table S2).

To mitigate the risk of overfitting, we applied LASSO regression for variable selection (Supplementary Figure S2 and S3). The core variables identified-ln(NLR), dialysis vintage, albumin, and CRP-were used to construct a simplified model. The effect estimate for ln(NLR) remained consistent in direction with that of the fully adjusted model (simplified model OR = 7.31, 95% CI: 3.05–17.52, *p* < 0.001; Supplementary Table S3), supporting the robustness of the primary finding. Collectively, these sensitivity analyses reinforce the conclusion that an elevated pre-existing NLR is a robust risk factor for subsequent PDAP in this patient population.

## 4 Discussion

The principal finding of this retrospective case–control study is that a higher neutrophil-to-lymphocyte ratio (NLR), measured up to 3 months prior to clinical presentation, is independently associated with an increased risk of developing peritoneal dialysis-associated peritonitis (PDAP). This association demonstrated robustness across multiple analytical approaches: it remained significant in multivariable logistic regression models with progressive adjustment for potential confounders, exhibited a significant positive linear dose–response relationship in curve fitting analysis, and was confirmed in complementary sensitivity analyses.

The NLR is an emerging, readily accessible biomarker of systemic inflammation. Its prognostic utility extends beyond dialysis populations, with established links to conditions such as contrast-induced nephropathy following vascular procedures (7). In the context of end-stage renal disease, elevated NLR has been consistently associated with heightened inflammatory burden, increased mortality, and adverse cardiovascular outcomes in both hemodialysis (HD) and peritoneal dialysis (PD) patients (3, 7, 8–15). Furthermore, an elevated NLR at the time of peritonitis diagnosis has been linked to treatment failure (4). Complementary evidence from other HD cohorts further supports the prognostic value of NLR as an independent predictor of mortality, with its significance persisting across different dialysis-vintage strata (16). Among patients undergoing PD and younger than 60 years, NLR was identified as an independent risk factor for poor cardiovascular outcomes (17). Additionally, another study demonstrated that an elevated NLR was a significant risk factor for developing pneumonia in patients undergoing PD, with multivariate Cox proportional hazards models confirming NLR as an independent predictor (18). Our study extends this evidence by demonstrating, for the first time, that a pre-morbid elevation in NLR-assessed during a clinically stable period preceding the infection-serves as a significant risk factor for the subsequent development of PDAP itself. This suggests that a subclinical pro-inflammatory state, reflected by an elevated NLR, may predispose patients to infectious complications. Within this context, our study is the first to demonstrate that the NLR measured 3 months before the onset of peritonitis is a risk factor for peritonitis in patients undergoing PD.

Current guidelines and the existing literature have identified a broad spectrum of risk factors for PDAP, encompassing patient demographics (e.g., age, diabetes), procedural aspects (e.g., training duration, aseptic technique), and dialysis-related factors (e.g., modality, solution storage) (19–21). Our findings introduce pre-infection NLR as a novel, biologically plausible candidate within this risk profile. The observed association remained robust after adjustment for several of these established factors, including dialysis vintage and nutritional markers such as albumin and globulin, suggesting its potential as an independent contributor to peritonitis risk.

Our findings corroborate and extend the growing literature on the prognostic value of the neutrophil-to-lymphocyte ratio (NLR) in the peritoneal dialysis (PD) population. Previous work showed that NLR measured after PD catheterization independently predicts subsequent PD-associated peritonitis (PDAP) with strong discrimination (22). In contrast, we demonstrate for the first time that NLR elevation can precede clinical infection, detectable during a stable, infection-free period up to 3 months before PDAP onset, supporting its role as a predisposing risk factor rather than solely an acute-phase marker.

Beyond NLR, inflammatory-nutritional markers have been linked to PDAP risk in other work. Nomograms incorporating lymphocyte count, albumin, and hemoglobin have achieved high predictive performance (AUC ≈ 0.93, Wang et al. 23). Lymphopenia, a key component of elevated NLR, and related nutritional risks have repeatedly correlated with infection risk, with large retrospective cohorts (You et al. 24) confirming that low albumin and low hemoglobin are PDAP risk factors alongside socioeconomic and lifestyle factors. These findings across designs and populations strengthen the biological plausibility that systemic inflammation and nutritional status influence PDAP susceptibility. Differences in timing of NLR measurement, population characteristics, cutoff choices, and analytic approaches likely explain heterogeneity in effect sizes. Prospective validation in diverse cohorts and standardization of measurement protocols will be essential before clinical implementation.

Several limitations of this retrospective case–control study should be acknowledged. First, although we used pre-peritonitis laboratory values to establish a temporal relationship between NLR and subsequent PDAP risk, reverse causation cannot be entirely excluded, as subclinical or prodromal infection may elevate NLR before diagnosis, despite excluding tests after symptom onset and performing sensitivity analyses restricting measurements to >30 days before the event. Prospective cohorts with baseline NLR at dialysis initiation are needed to better establish temporal precedence. Second, despite rigorous matching, blinded data extraction, and adjustment for known confounders, residual confounding from unmeasured factors-such as detailed comorbidity scores, medication use, socioeconomic status, and treatment adherence-may influence the observed associations. Third, NLR was measured at a single time point, which may not capture longitudinal fluctuations in inflammatory status; serial measurements could provide a more complete inflammatory trajectory and potentially improve PDAP risk prediction. Fourth, modest sample sizes in some subgroup analyses yielded wide confidence intervals and unstable estimates (for example, an OR of 47.28 in patients without hypertension). These exploratory findings require validation in larger, adequately powered studies. Fifth, the ROC analysis for NLR alone was exploratory; we did not compare NLR to a baseline clinical model, assess incremental predictive value, evaluate calibration, or perform internal validation. Consequently, while NLR showed moderate discrimination (AUC 0.78), conclusions about its clinical utility for risk stratification remain preliminary, and external validation in large, prospective multicenter cohorts is necessary before clinical implementation. Finally, selection bias may exist because patients with incomplete medical records were excluded, and the single-center design with a relatively modest sample size may limit generalizability and precision of effect estimates; multicenter studies are needed to validate results and explore potential effect modification across diverse populations and settings.

In conclusion, our study provides evidence that an elevated neutrophil-to-lymphocyte ratio measured during a stable clinical period is associated with a significantly increased risk of subsequent peritonitis in patients undergoing peritoneal dialysis. If validated in larger, prospective multicenter cohorts, the NLR could serve as a simple and inexpensive tool for risk stratification, potentially identifying patients who may benefit from intensified monitoring or targeted preventive strategies. Future research should aim to confirm this association, elucidate the underlying mechanisms linking systemic inflammation to peritoneal infection risk, and explore the integrated predictive value of NLR alongside traditional and novel risk factors.

## Data Availability

The raw data supporting the conclusions of this article will be made available by the authors, without undue reservation.

## Supplementary material

Supplementary FIGURE S1 Receiver operating characteristic (ROC) curve evaluating the predictive performance of NLR for peritonitis.

Supplementary FIGURE S2 LASSO Logistic Regression for Feature Selection. Selection of the optimal penalization parameter (lambda) in the LASSO model using five-fold cross-validation based on the minimum deviance criterion.

Supplementary FIGURE S3 LASSO Logistic Regression for Feature Selection. LASSO coefficient paths for the 14 candidate features. Coefficient profiles are plotted as a function of the log(lambda) sequence. The vertical dashed line indicates the optimal lambda value selected by five-fold cross-validation, at which four features retained non-zero coefficients. LASSO, Least Absolute Shrinkage and Selection Operator.

Supplementary Table S1 Risk factors for peritoneal dialysis-related peritonitis.

Supplementary Table S2 ROC analysis for NLR in predicting peritoneal dialysis-associated peritonitis.

Supplementary Table S3 Simplified multivariable logistic regression model for the association between NLR and peritoneal dialysis-associated peritonitis.
