# Supplementary figures and images for: Neutrophil-to-lymphocyte ratio as a harbinger of peritonitis in peritoneal dialysis: a case–control study
Source: Front Med (Lausanne). 2026 Apr 16;13:1787005. doi: 10.3389/fmed.2026.1787005 (PMC13128413; doi:10.3389/fmed.2026.1787005)

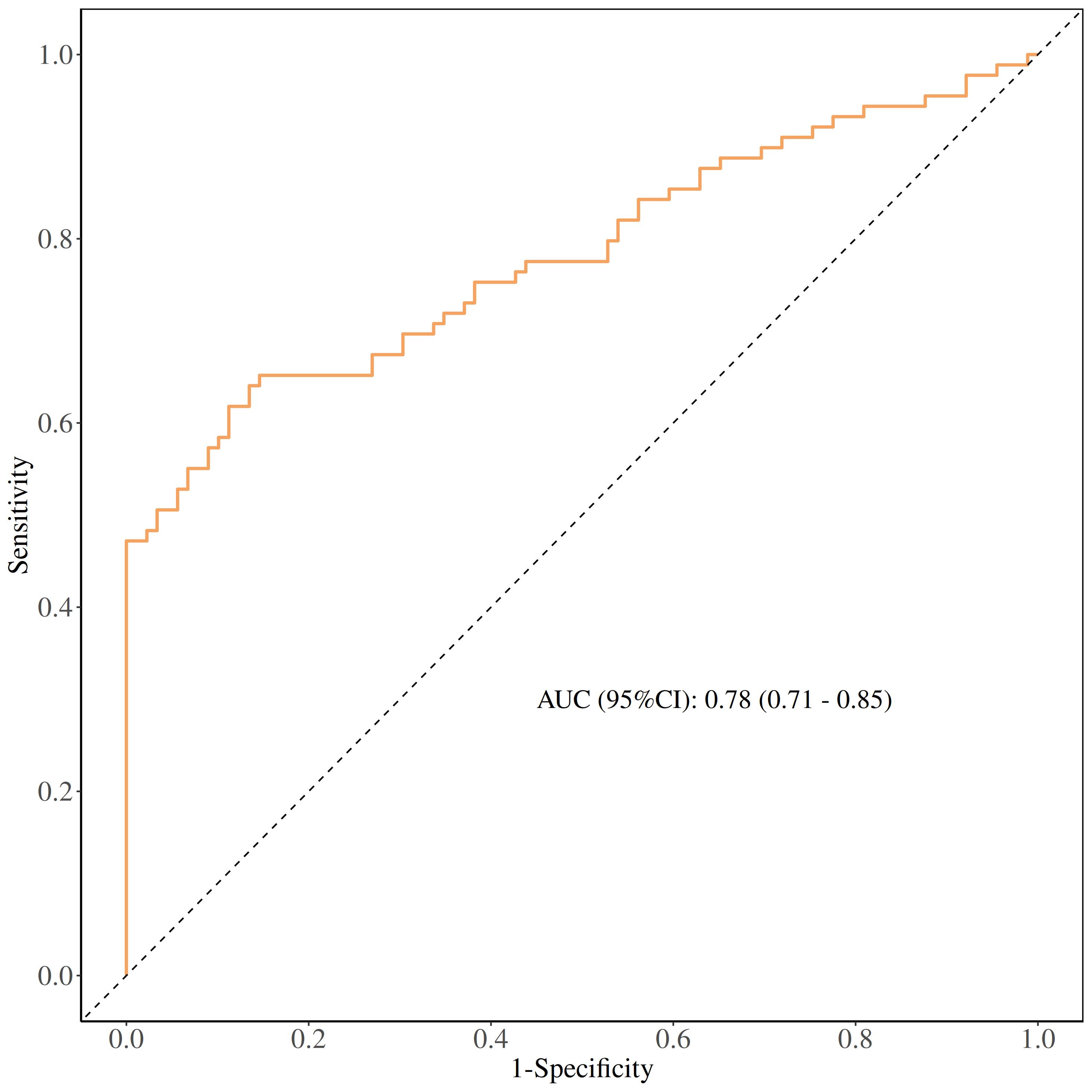

Supplement: Supplementary FIGURE S1 — Receiver operating characteristic (ROC) curve evaluating the predictive performance of NLR for peritonitis. [file Image_1.TIFF]

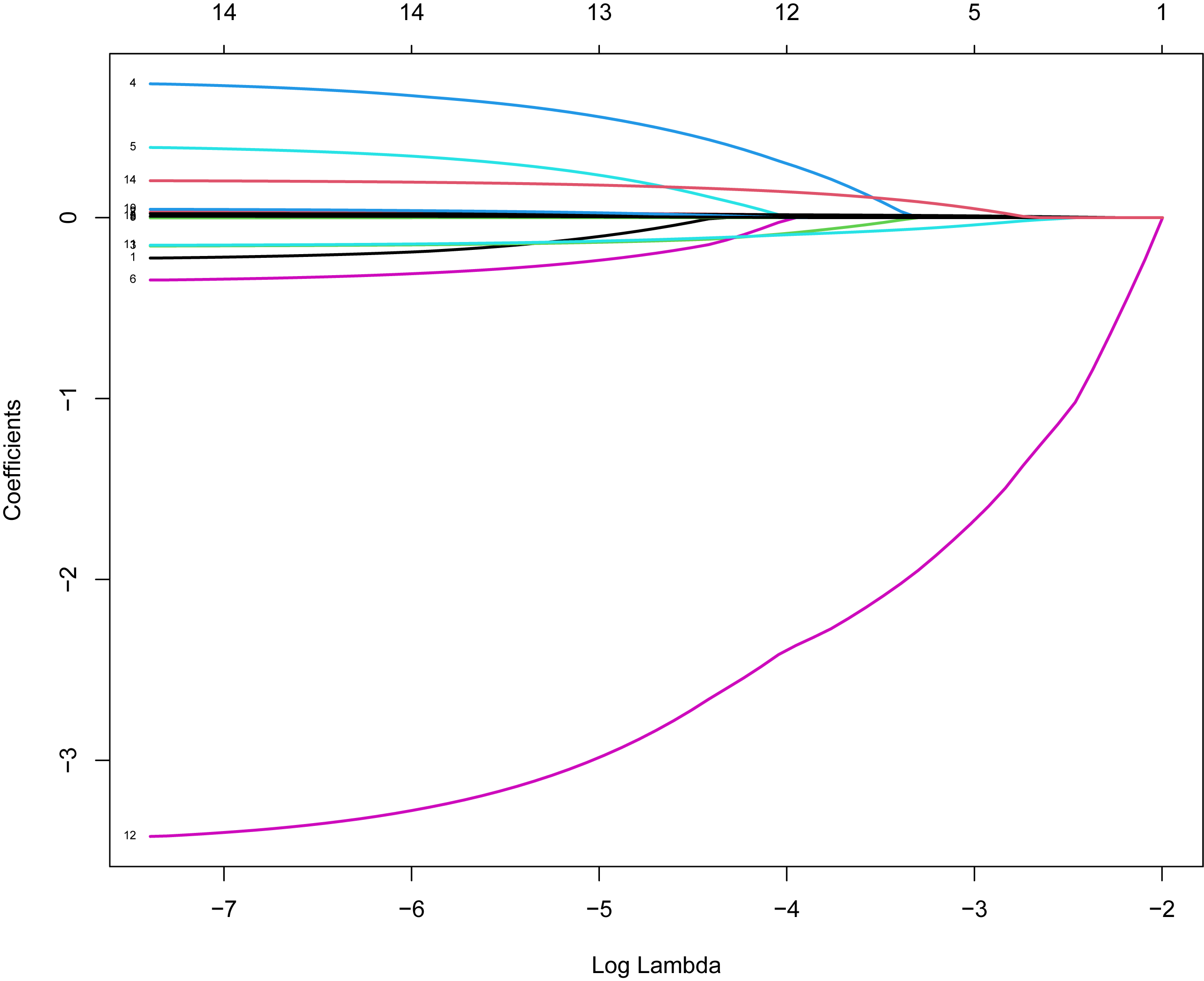

Supplement: Supplementary FIGURE S2 — LASSO Logistic Regression for Feature Selection. Selection of the optimal penalization parameter (lambda) in the LASSO model using five-fold cross-validation based on the minimum deviance criterion. [file Image_2.TIF]

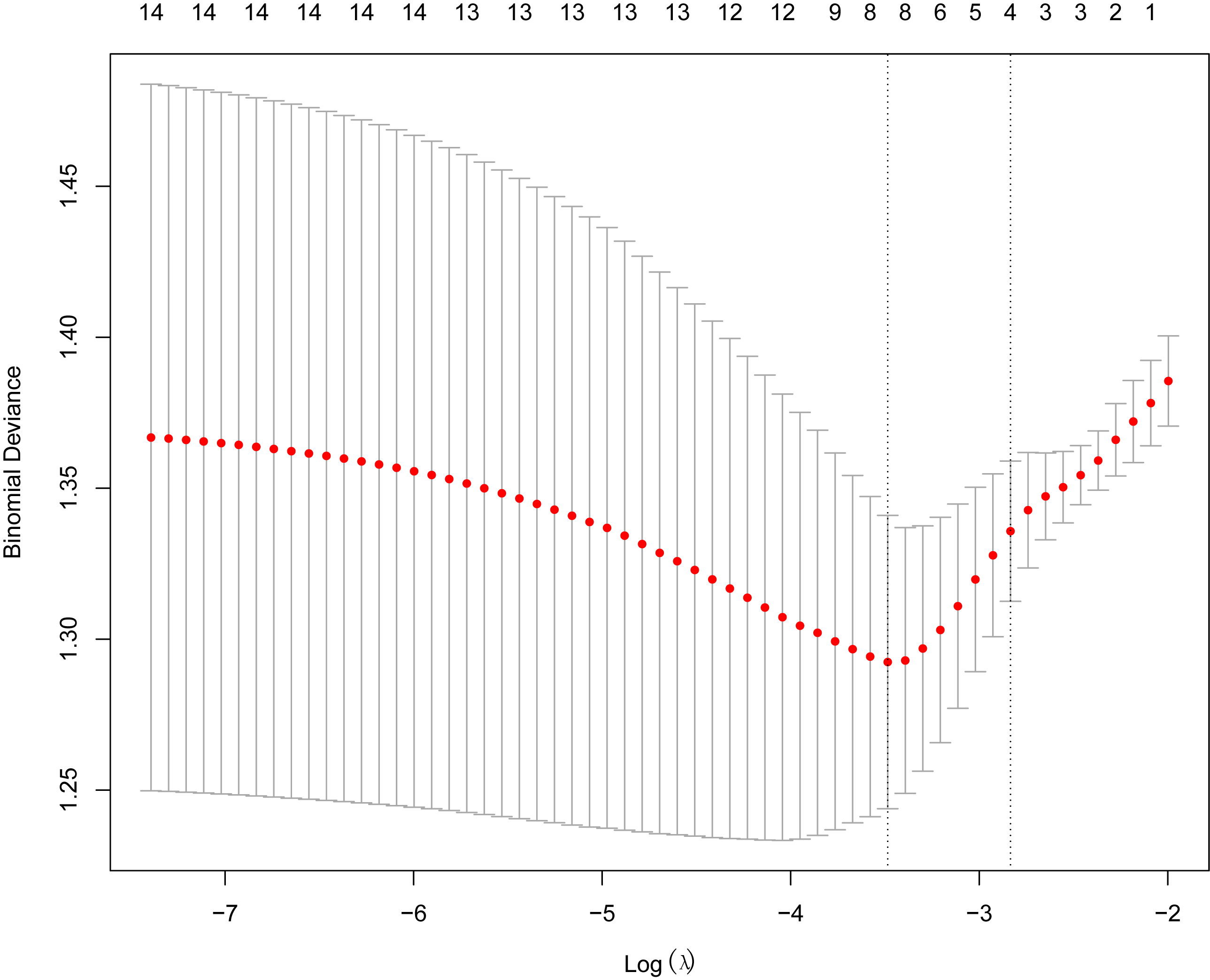

Supplement: Supplementary FIGURE S3 — LASSO Logistic Regression for Feature Selection. LASSO coefficient paths for the 14 candidate features. Coefficient profiles are plotted as a function of the log(lambda) sequence. The vertical dashed line indicates the optimal lambda value selected by five-fold cross-validation, at which four features retained non-zero coefficients. LASSO, Least Absolute Shrinkage and Selection Operator. [file Image_3.TIF]
