# Supplementary material for: Neutrophil-to-lymphocyte ratio as a harbinger of peritonitis in peritoneal dialysis: a case–control study
Source: Front Med (Lausanne). 2026 Apr 16;13:1787005. doi: 10.3389/fmed.2026.1787005 (PMC13128413; doi:10.3389/fmed.2026.1787005)
Supplement: Supplementary Table S1 — Risk factors for peritoneal dialysis-related peritonitis. [file Table_1.DOCX]

**Table S1. Risk Factors for Peritoneal Dialysis-Related Peritonitis**

| Variable | Bivariate Analysis |  | Multivariate Analysis |  |
| --- | --- | --- | --- | --- |
|  | OR (95% CI) | *P Value* | Adjusted OR (95% CI) | *P Value* |
| NLR (per 1-unit increase) | 9.45 (3.89 – 22.96) | <0.001 | 3.01 (1.23 – 7.33) | 0.015 |
| Dialysis Duration (per month) | 1.02 (1.01 – 1.03) | <0.001 | 1.02 (1.01 – 1.03) | 0.015 |
| Kt/V |  |  |  |  |
| < 1.7 | 1.00 (Reference) |  | 1.00 (Reference) |  |
| ≥ 1.7 | 0.47 (0.18 – 1.25) | 0.131 | 0.40 (0.13 – 1.27) | 0.121 |
| Calcium (mmol/L) |  |  |  |  |
| < 2.0 | 1.00 (Reference) |  | 1.00 (Reference) |  |
| ≥ 2.0 | 0.23 (0.07 – 0.73) | 0.013 | 0.15 (0.04 – 0.57) | 0.005 |
| Globulin (per 1 g/L increase) | 0.92 (0.86 – 0.98) | 0.010 | 0.84 (0.75 – 0.93) | 0.002 |
| Albumin (per 1 g/L increase) | 0.96 (0.90 – 1.02) | 0.195 | 1.06 (0.96 – 1.17) | 0.279 |
| Diabetes |  |  |  |  |
| No | 1.00 (Reference) |  | 1.00 (Reference) |  |
| Yes | 2.39 (0.71 – 8.07) | 0.160 | 1.66 (0.36 – 7.63) | 0.516 |

**Footnotes:**
The multivariate logistic regression model included all variables with P < 0.20 from the bivariate analysis. The following variables were analyzed in the bivariate analysis but did not meet the inclusion threshold (P ≥ 0.20) and were thus not included in the multivariate model: Hemoglobin (<110 vs. ≥110 g/L) and Smoking (Yes vs. No).Abbreviations: CI, confidence interval; NLR, neutrophil-to-lymphocyte ratio; OR, odds ratio.
