# Supplementary material for: Neutrophil-to-lymphocyte ratio as a harbinger of peritonitis in peritoneal dialysis: a case–control study
Source: Front Med (Lausanne). 2026 Apr 16;13:1787005. doi: 10.3389/fmed.2026.1787005 (PMC13128413; doi:10.3389/fmed.2026.1787005)
Supplement: Supplementary Table S2 — ROC analysis for NLR in predicting peritoneal dialysis-associated peritonitis. [file Table_2.DOCX]

| **AUC**  **(95%CI)** | **Accuracy**  **(95%CI)** | **Sensitivity**  **(95%CI)** | **Specificity**  **(95%CI)** | **PPV (95%CI)** | **NPV**  **(95%CI)** | **Cut off** |
| --- | --- | --- | --- | --- | --- | --- |
|  |  |  |  |  |  |  |
| 0.78 (0.71-0.85) | 0.75 (0.68-0.81) | 0.85 (0.78 - 0.93) | 0.65 (0.55 - 0.75) | 0.71 (0.62 - 0.80) | 0.82 (0.73 - 0.91) | 0.459 |

TableS2. ROC analysis for NLR in predicting Peritoneal Dialysis–associated Peritonitis
