# Supplementary material for: Neutrophil-to-lymphocyte ratio as a harbinger of peritonitis in peritoneal dialysis: a case–control study
Source: Front Med (Lausanne). 2026 Apr 16;13:1787005. doi: 10.3389/fmed.2026.1787005 (PMC13128413; doi:10.3389/fmed.2026.1787005)
Supplement: Supplementary Table S3 — Simplified multivariable logistic regression model for the association between NLR and peritoneal dialysis-associated peritonitis. [file Table_3.DOCX]

**Table S3. Simplified Multivariable Logistic Regression Model for the Association Between NLR and Peritoneal Dialysis-Associated Peritonitis**

| **Variable** | **Multivariate Analysis** |  |
| --- | --- | --- |
|  | **Adjusted OR (95% CI)** | ***P Value*** |
| NLR (per 1-unit increase) | 7.31 (3.05~17.52) | <0.001 |

**Footnotes:**

The simplified model was adjusted for core variables selected via LASSO regression, including ln(NLR), dialysis vintage, albumin, and C-reactive protein (CRP). Abbreviations: CI, confidence interval; NLR, neutrophil-to-lymphocyte ratio; OR, odds ratio.
